# Digging deeper into NINJ1: its multifaceted role in central nervous system diseases

**DOI:** 10.3389/fimmu.2026.1838013

**Published:** 2026-05-29

**Authors:** Mengting Tian, Meicen Zhou, Shiping Li, Yi Qu

**Affiliations:** 1 Department of Pediatrics, West China Second University Hospital, Sichuan University, Chengdu, Sichuan, China; 2 Key Laboratory of Birth Defects and Related Diseases of Women and Children, Sichuan University, Ministry of Education, Chengdu, Sichuan, China

**Keywords:** central nervous system, lytic cell death, neuroinflammation, *Ninj1*, plasma membrane rupture

## Abstract

Ninjurin1 (NINJ1) is a cell-surface molecule that has gained considerable attention for its role in mediating plasma membrane rupture (PMR). Originally identified as an adhesion molecule induced after nerve injury, NINJ1 is now recognized as a common terminal executor of PMR across multiple forms of lytic cell death, including pyroptosis, necroptosis, and ferroptosis. This function positions NINJ1 as a key link between cell death and inflammatory activation. However, the precise role of NINJ1 in the central nervous system (CNS) remains unclear. This review systematically outlines the molecular structure, expression, activation, and regulation of NINJ1, with a focus on its multifaceted roles in CNS disorders, including multiple sclerosis, ischemic stroke, traumatic brain injury, spinal cord injury, neuropsychiatric disorders and neurodegenerative diseases. We also highlight critical knowledge gaps, particularly regarding cell type–specific functions in the CNS. Finally, we evaluate therapeutic strategies targeting NINJ1 (including monoclonal antibodies, functional peptides, and small-molecule inhibitors) and their potential applications in neurological diseases. By integrating current evidence and identifying unresolved questions, this review aims to provide a foundation for future mechanistic and translational studies of NINJ1 in the CNS.

## 1 Introduction

Lytic cell death, such as pyroptosis, necroptosis, and ferroptosis, is characterized by the progressive loss of plasma membrane integrity and the uncontrolled release of damage-associated molecular patterns (DAMPs) that drive inflammation (1). For decades, the final step in plasma membrane rupture (PMR) has been considered a passive osmotic consequence of cell swelling. This view was fundamentally revised by the discovery that Ninjurin1 (NINJ1) acts as an active, regulated executor of PMR in multiple lytic death pathways (2).

Accumulating evidence has established a strong link between lytic cell death and the pathogenesis of central nervous system (CNS) disorders, such as multiple sclerosis (MS), traumatic brain injury (TBI) and spinal cord injury (SCI) (3, 4). In this context, NINJ1 has emerged as a key downstream effector mediating PMR and the subsequent DAMP release that fuels neuroinflammation (5–7). Importantly, NINJ1 was originally identified as an adhesion molecule upregulated in neurons and Schwann cells after nerve injury (8). Later studies, particularly in experimental autoimmune encephalomyelitis (EAE), revealed its critical role in immune cell adhesion, migration, and transendothelial infiltration into the CNS (9–11). Thus, NINJ1 possesses a dual nature: it contributes to both the execution of lytic cell death and the regulation of neuroinflammatory cell trafficking, making it a uniquely versatile player in CNS pathology.

Recent advances in structural biology have resolved the three-dimensional architecture of NINJ1, uncovering its autoinhibitory mechanism, the conformational changes that drive oligomerization, and the distinct “cut-and-release” model of membrane disruption (2, 12–15). These structural insights, together with the identification of upstream activation signals (mechanical tension, calcium, and lipid peroxidation) and long-term expression regulators (p53, miR-125a-5p, etc.), have greatly expanded our understanding of how NINJ1 is controlled and how it executes its functions (16–19). However, the precise roles of NINJ1 in different CNS diseases and the therapeutic implications of targeting it remain to be systematically synthesized.

In this review, we integrate the current knowledge on NINJ1 based on its molecular structure, the lytic cell death pathways in which it operates, its tissue distribution, biological functions, and regulatory mechanisms. We then examine the evidence for NINJ1 in CNS disorders (MS, ischemic stroke, TBI, SCI, neuropsychiatric disorders, and neurodegenerative diseases) and highlight the critical knowledge gaps, particularly regarding cell type–specific functions. Finally, we evaluate existing therapeutic strategies targeting NINJ1 and outline future directions. By providing a comprehensive and critical synthesis, this review aims to guide future mechanistic and translational studies of NINJ1 in the CNS.

## 2 Structure of NINJ1

NINJ1 is a 16-kDa transmembrane protein whose cDNA was first identified in 1997 and mapped to human chromosome 9q22 (20). Its amino acid sequence exhibits a high degree of evolutionary conservation across species; sequence alignment shows 89.5% identity and 95.4% homology between mouse and human NINJ1, with broader conservation maintained among vertebrates (12, 21). The human NINJ1 gene encodes a 152-amino acid polypeptide that features two transmembrane helices (positioned at amino acids 72–100 and 118–139), an intracellular region, and two extracellular segments at the N- and C-termini (21, 22).

Notably, the N-terminal segment contains a critical functional domain: a 12-residue stretch from Pro26 to Asn37 characterized by tryptophan and a contiguous cluster of arginine residues that mediate homophilic and heterophilic adhesion (20, 21, 23). Among which, the 29th tryptophan residue and three arginine residues in this region are particularly important (20). After mutation of these residues, the adhesive function of NINJ1 was significantly inhibited. Furthermore, studies have revealed that NINJ1 assembles into homomeric complexes through homophilic interactions, and this assembly is maintained by N-glycosylation at Asn60 and an intracellular region spanning Leu101–Ala110 (21). It has been demonstrated that NINJ1 acts as a cis-interacting protein and exists as a homomeric complex in living cells. When the Asn60 residue is mutated via site-directed mutagenesis or when cells are treated with glycosylation inhibitors, the glycosylation of NINJ1 is disrupted. This results in a significant reduction in dimer and oligomer formation, increased susceptibility to proteolytic degradation, decreased protein stability, and impaired adhesive function.

**Figure 1:**
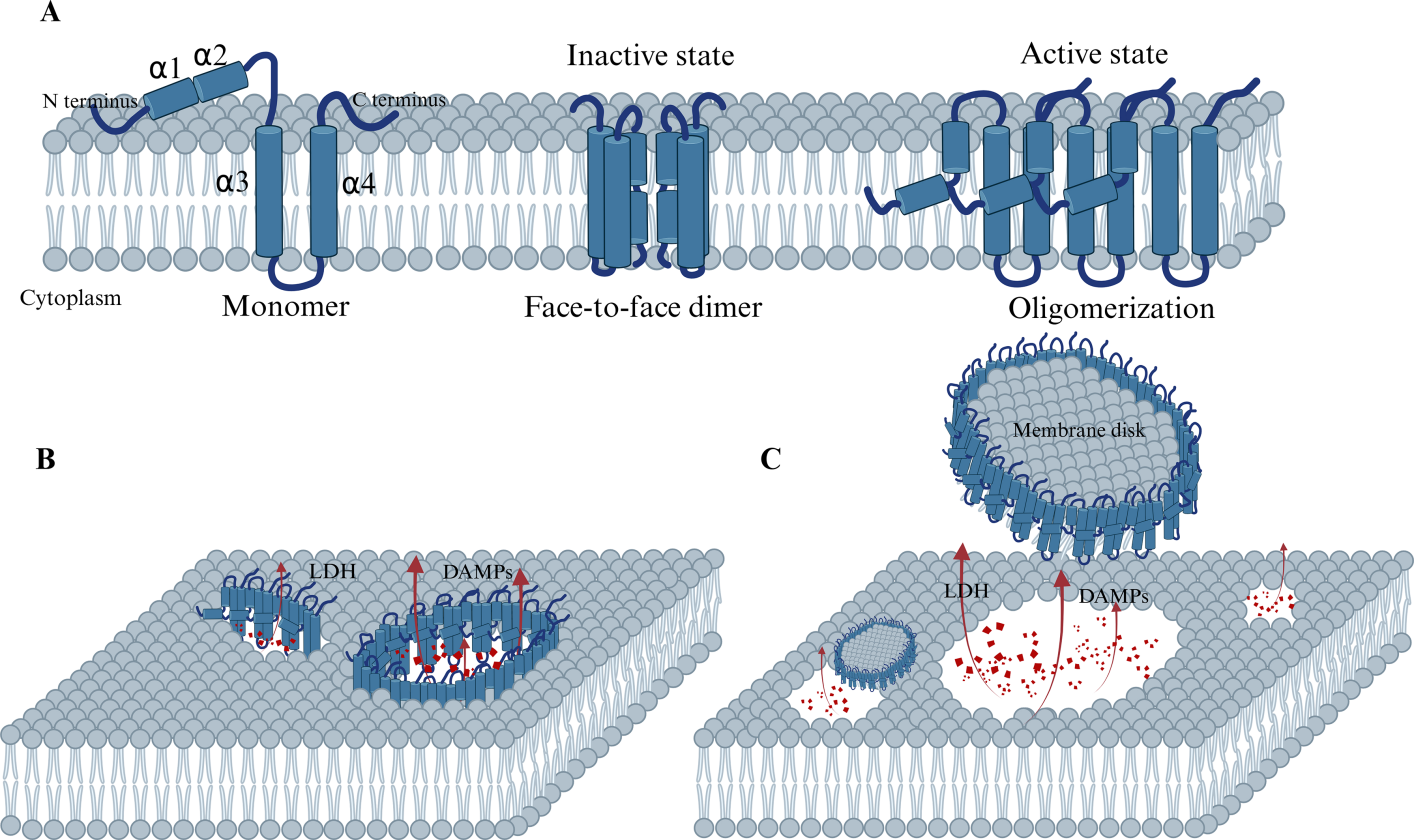
The mechanism of NINJ1 mediated PMR. **(A)** NINJ1 exists as an inactive monomer or an autoinhibited “face-to-face” dimer. Upon cell death signals, it undergoes a conformational change and initiates ordered oligomerization via head-to-head interactions of the α1 helix. **(B)** Filament model of NINJ1-dependent PMR proposed by Degen et al. Activated NINJ1 oligomerizes into filaments, ultimately mediated PMR. **(C)** Cookie-cutter model of NINJ1-dependent PMR proposed by David et al. NINJ1 oligomerizes into irregular rings that cut membrane patches within rings and releases membrane disks to mediate PMR. [The image was created using BioRender (https://app.biorender.com/illustrations/6944a3ce279955ae3522002d?slideId=48361938-03d3-442f-9ba0-9e7e0c86b644)].

With the application of advanced structural biology techniques such as cryo-electron microscopy, the three-dimensional architecture of NINJ1 has been precisely depicted (12). Degen et al. revealed that the first 38 N-terminal amino acids of NINJ1 remained disordered, while the subsequent amino acids (39–141) fold to form four well-defined α-helices, named α1 to α4. Among these, the α3 and α4 helices serve as membrane anchors, whereas the α1 helix functions as the core functional unit responsible for oligomerization and membrane insertion. When NINJ1 exists as a monomer anchored to the plasma membrane, its α1 and α2 helices extend into the extracellular space, while the α3 and α4 helices embed within the lipid bilayer, forming the transmembrane domain (**Figure 1A**). Upon detection of cell death signals, such as inflammasome activation, NINJ1 undergoes a conformational change and initiates an ordered oligomerization process (**Figure 1A**). Within 10 minutes, NINJ1 monomers engage in head-to-head homotypic interactions via the α1 helix, assembling into large-scale filamentous structure. These fibrils are inserted into the lipid bilayer, forming dimeric and trimeric intermediates that rapidly organize into extensively branched filamentous superstructures (**Figure 1B**). Ultimately, this disruption leads to the formation of fissures or pores in the membrane, resulting in PMR.

However, regarding the mechanism of NINJ1-mediated PMR, David et al. propose a distinct “cookie-cutter” model (13) (**Figure 1C**). Using live-cell and super-resolution imaging, they observed that numerous membrane discs were released from the cell surface during PMR. The progressive release of membrane discs generates substantial gaps in the membrane, leading to cytoplasmic leakage and eventual cellular disintegration. Consequently, this process enables a more complete release of DAMPs, establishing that NINJ1 mediates PMR through a novel “cut-and-release” mechanism. Additionally, Pourmal et al. (14) identified an autoinhibitory mechanism of NINJ1. In its inactive state, NINJ1 adopts an unkinked three-helix conformation and assembles into a “face-to-face” dimer, in which the N-terminal functional domains of both monomers are mutually locked at the dimer interface (**Figure 1A**). Upon initiation of cell death, structural alterations in the plasma membrane release this autoinhibition, freeing the N-terminal domains to drive NINJ1 oligomerization and execute membrane rupture.

## 3 Expression and distribution of NINJ1

NINJ1 was initially identified as a novel injury-induced molecule in neurons and Schwann cells (8) and subsequent studies revealed its widespread expression in both embryonic and adult rats, extending beyond the nervous system (20), suggesting its involvement in broader biological processes. In 2019, Ekanayake et al. (24) further confirmed the localization of NINJ1 in various tissues, including the cerebrum, sciatic nerve, spleen, lung, stomach, ileum, colon, liver, pancreas, kidney, testis, and skin in C57BL/6 mice. These findings indicate that NINJ1 is not merely an “emergency” protein induced upon injury, but is constitutively and selectively expressed under physiological conditions across diverse tissues and cell types. Its expression profile suggests a potential role in maintaining cellular adhesion, tissue homeostasis, and basic immune function in multiple organ systems.

Under various pathological conditions, NINJ1 expression undergoes dynamic alterations. NINJ1 expression is upregulated in MS (10), cerebral ischemia (25), intracerebral hemorrhage (5), non-small cell lung cancer (26), retroperitoneal liposarcoma (27), thoracic aortic dissection (28), abdominal aortic aneurysm (29), diabetic vascular complication (30, 31), retinal ischemia/reperfusion injury (32, 33), liver ischemia/reperfusion injury (34), liver failure (35), pulmonary fibrosis (36), intestinal inflammatory conditions (37), pancreatitis (38), SCI (39), cavernous nerve injury (40), gout flares (41), acute kidney injury (42) and sepsis-associated disseminated intravascular coagulation (43), suggesting its involvement in the pathogenesis of these conditions. In addition to its tissue-specific expression, circulating NINJ1 levels have emerged as biomarkers. Elevated plasma NINJ1 levels have been reported in patients with sepsis (44), large artery atherosclerotic acute ischemic stroke (45), atrial fibrillation (46), coronary artery disease (47), systemic lupus erythematosus (48), coronavirus disease 2019 (49) and hepatocellular carcinoma (50), and its levels often correlate with disease severity.

## 4 Function of NINJ1

### 4.1 Basic biological and physiological functions

NINJ1 was initially identified as a cell adhesion molecule upregulated in neurons and Schwann cells following nerve injury (8). It mediates homotypic adhesive interactions between neurons and Schwann cells via its N-terminal domain, providing essential contact guidance and signaling for axonal regeneration, thereby promoting nerve repair. Subsequent studies revealed that NINJ1’s adhesive function is universal; it can also induce strong intercellular aggregation, even in typically non-adherent cells such as L929 fibroblasts (20). In addition to its role in cell adhesion, NINJ1 also plays a role in cell chemotaxis and migration. Its N-terminal fragment can be cleaved by matrix metalloproteinase-9 (MMP9), and this fragment exhibits chemotactic activity by directly attracting monocytes/macrophages and neutrophils (51). Furthermore, in endothelial cell experiments, the N-terminal fragment exerted its pro-angiogenic effect by inhibiting endogenous NINJ1, thereby enhancing tube-forming capacity (23). NINJ1 activates the Ras-related C3 botulinum toxin substrate 1 (Rac1) signaling pathway, induces actin reorganization and pseudopodia formation, and directly enhances the motility and transendothelial migration ability of immune cells, such as monocytes (9, 11). Biological functions determine the crucial role of NINJ1 in maintaining tissue homeostasis. For example, NINJ1 has been shown to contribute to myocyte growth and differentiation (52). Following peripheral nerve injury, NINJ1 is crucial for promoting the participation of NG2-positive Schwann cells in the myelination of regenerating nerves (53). In pericytes, NINJ1 contributes to vasa vasorum maturation in response to vascular injury and reduces vascular remodeling (54). Furthermore, NINJ1 interferes with the activation of interleukin 6 and the signal transducer and activator of transcription 3 (STAT3) signaling pathways, thereby inhibiting the migration, invasion, and metastatic potential of lung cancer cells (55). Intriguingly, research has revealed that NINJ1 can oligomerize on the surface of large apoptotic bodies, where it regulates vesicle stability and controls the release of DAMPs and noroviruses from these vesicles. This finding suggests that NINJ1 may act as a “regulator” of membrane and its derivative stability, with its functional outcome depending on the specific membrane environment and cellular context (56). Additionally, NINJ1 plays a protective role by maintaining intestinal immune homeostasis and microbial balance. Its deficiency exacerbates colitis development by promoting macrophage polarization towards a pro-inflammatory M1 phenotype and inducing microbial imbalance (57). *Ninj1* knockout mice also exhibit significant behavioral abnormalities such as repetitive and anxiety-like behaviors, indicating that NINJ1 may finely regulate behavior through neuroimmune interactions in key brain regions (58).

### 4.2 Promoting inflammation

When NINJ1 expression or function is dysregulated, its core adhesion and migration properties become key drivers of disease, with its chemotactic and adhesive activities serving as potent inflammatory amplifiers in multiple disease models. NINJ1 enhances the c-Jun N-terminal kinase and p38 pathways by suppressing dual-specificity phosphatase 1, promoting macrophage release of chemokines such as C-X-C motif chemokine ligand 1 (CXCL1), which subsequently recruits large numbers of neutrophils and amplifies inflammatory responses (34). Upon lipopolysaccharide (LPS) stimulation, NINJ1 exacerbates inflammation by promoting the recruitment of myeloid cells to inflammatory sites and enhancing their pro-inflammatory activity (37). In a septic mouse model, repression of NINJ1 by an inhibitory peptide or small interfering RNA attenuated LPS-triggered inflammation in macrophages and endothelial cells by modulating p38 phosphorylation and activator protein-1 activation (59).

**Figure 2:**
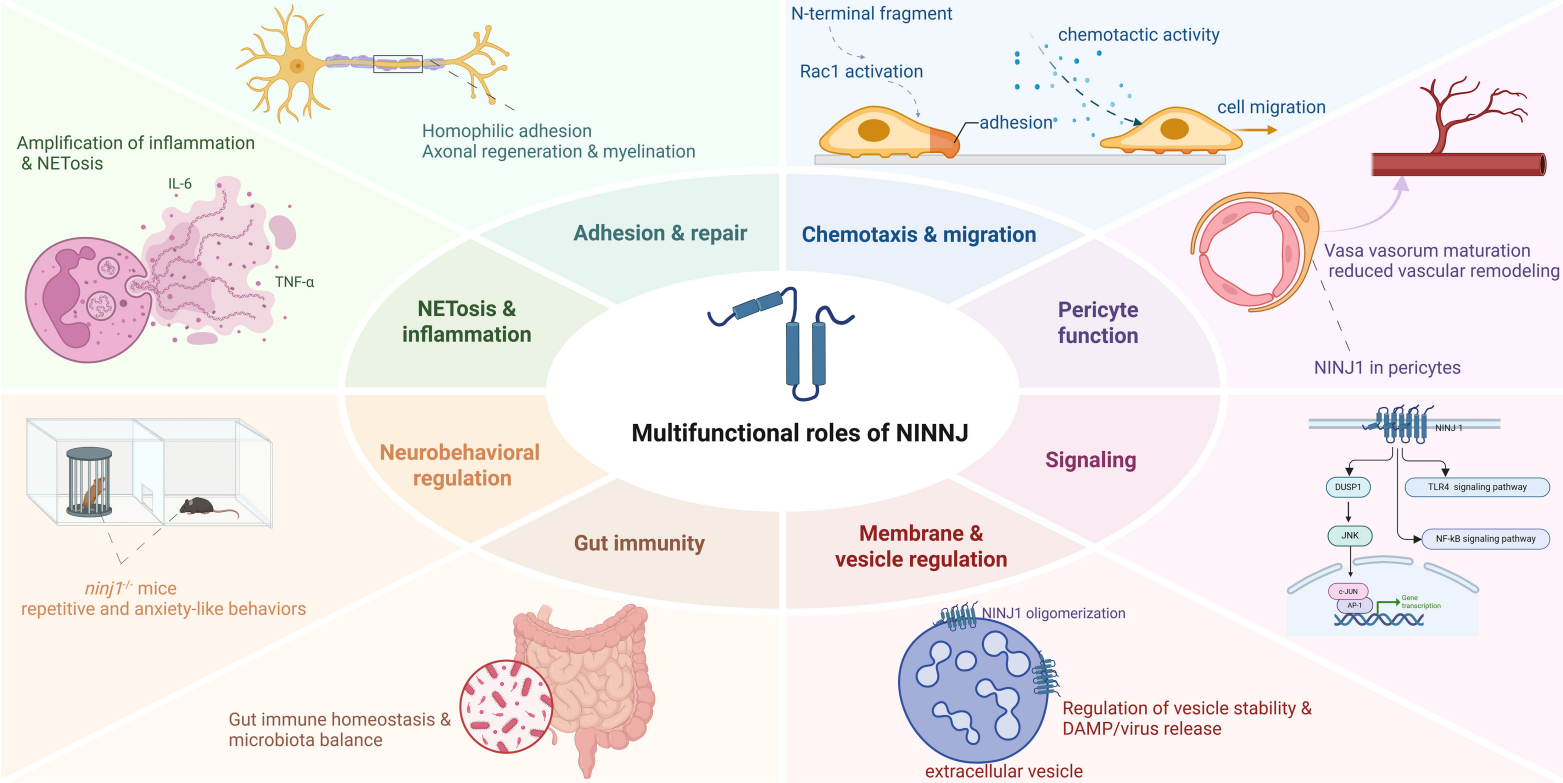
Pleiotropic functions of NINJ1 beyond plasma membrane rupture. NINJ1 participates in various biological processes independent of its role in PMR, including: (1) Cell adhesion and nerve repair – homotypic adhesion between neurons and Schwann cells promoting axonal regeneration and myelination; (2) Chemotaxis and migration – N-terminal fragment attracts monocytes/macrophages/neutrophils, activates Rac1, and enhances transendothelial migration; (3) Vascular remodeling – in pericytes, NINJ1 reduces vascular remodeling and promotes vasa vasorum maturation after injury; (4) Signaling pathways – mediates signaling pathways such as JNK/p38, NF-κB, and TLR4; (5) Membrane and vesicle regulation – oligomerizes on apoptotic bodies to control DAMP and norovirus release; (6) Gut immune homeostasis – regulates macrophage polarization (M1/M2) and microbial balance; (7) Neurobehavioral regulation – knockout mice exhibit anxiety and repetitive behaviors; (8) Inflammation amplification – promotes neutrophil extracellular trap formation. [The image was created using BioRender (https://app.biorender.com/illustrations/699d09d7a4fd366c20ac5642?slideId=bb2a206e-a38d-44a5-ad59-f4c1f9a954de)].

Furthermore, NINJ1 has been demonstrated to be involved in a range of diseases, including diabetes mellitus, endothelial dysfunction, gout, abdominal aortic aneurysm, and oxalate nephropathy, where it promotes cellular inflammation via multiple mechanisms. These include activating the p38 mitogen-activated protein kinase and nuclear factor kappa B (NF-κB) pathway (31), stimulating transendothelial transport proteins (30), activating Toll-like receptor 4 (TLR4) by competitively binding with Annexin A2 (29), and mediating neutrophil extracellular trap (NETs) formation in neutrophils (60). Collectively, these mechanisms sustain a self-amplifying inflammatory cycle. Notably, therapeutic interventions using NINJ1-knockout mice or anti-NINJ1 antibodies in these disease models significantly reduce inflammatory cytokine release and alleviate disease severity (29, 31, 41, 60). NINJ1 is a key molecular node that links inflammation with multiple forms of cell death. An overview of these NINJ1 functions is presented in **Figure 2**.

### 4.3 NINJ1 and lytic cell death pathways

**Figure 3:**
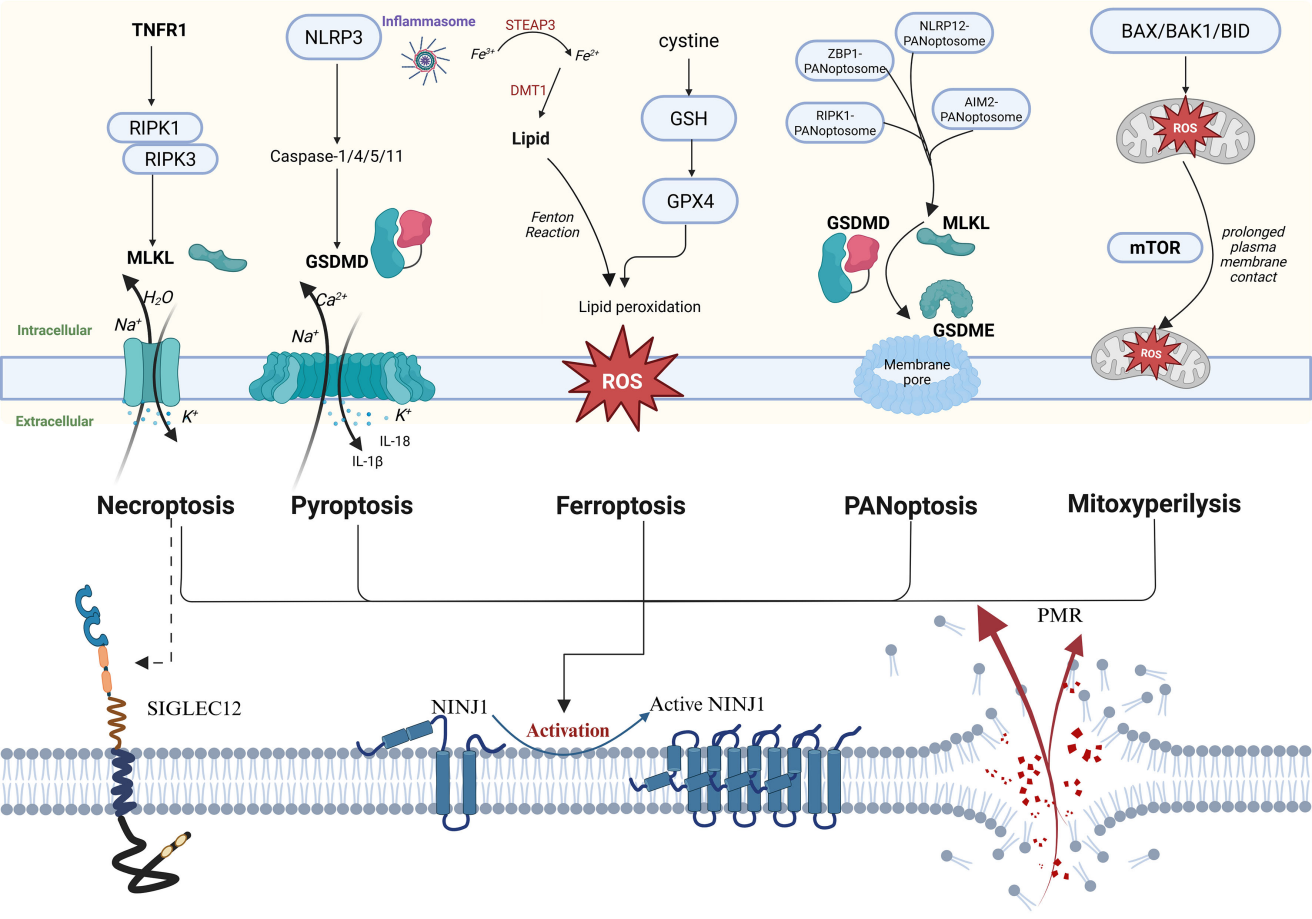
NINJ1 as a common terminal executor of plasma membrane rupture across lytic cell death pathways. NINJ1 operates downstream of these distinct lytic death pathways: pyroptosis (GSDMD-dependent), necroptosis (MLKL-dependent), ferroptosis (lipid peroxidation-driven), PANoptosis (PANoptosome-mediated), and mitoxyperilysis (mitochondrial ROS-induced). In necroptosis, SIGLEC12 provides an alternative PMR executor, indicating functional redundancy. [The image was created using BioRender (https://app.biorender.com/illustrations/69f9daf69459889ccb9be3bc?slideId=ae309b3a-54c4-4f19-8ef9-efba800ecf5e)].

NINJ1 has been established as a key terminal executor of lytic cell death, a process characterized by the loss of plasma membrane integrity and uncontrolled release of intracellular contents that drive inflammation. Lytic cell death encompasses several molecularly distinct pathways, including pyroptosis, necroptosis, ferroptosis, PANoptosis, and the recently identified mitoxyperilysis (1, 61–63). Despite their diverse triggers and initial execution mechanisms, these pathways converge in NINJ1-mediated PMR, positioning NINJ1 as a shared terminal checkpoint controlling DAMP release and subsequent inflammation. This section provides a concise overview of how NINJ1 participates in each of these death programs (**Figure 3**).

Pyroptosis is triggered by inflammasome activation, leading to caspase-1/4/5/11 mediated cleavage of gasdermin D (GSDMD) (64). The released N-terminal GSDMD fragment forms pores in the plasma membrane, eventually causing PMR. Kayagaki et al. (2) first demonstrated that NINJ1 oligomerization is required for the final membrane disintegration step in pyroptosis. In the absence of NINJ1, cells undergo GSDMD-dependent pore formation but fail to progress to complete PMR, resulting in reduced DAMP release. Thus, NINJ1 acts downstream of GSDMD to execute terminal lytic events.

Necroptosis is induced by the pseudokinase mixed lineage kinase domain-like protein (MLKL). Upon activation by RIPK1/RIPK3, MLKL oligomerizes and is inserted into the plasma membrane, disrupting its integrity (65). A seminal study by Kayagaki et al. first established a functional role for NINJ1 in necroptosis, but its role was neither essential nor exclusive, as *Ninj1* knockout only modestly affected MLKL-induced cell membrane rupture (2). More importantly, a recent study identified SIGLEC12 as an alternative PMR executor in necroptosis that can operate independently of NINJ1. Noh et al. demonstrated that SIGLEC12 mediates PMR, potentially through a similar filament−forming mechanism, and double deficiency of *Ninj1* and *Siglec12* exacerbates defects in membrane rupture (66). Therefore, NINJ1 participates in necroptotic PMR, it may serve as one of several alternative executors; further validation is required to determine their relative contributions, which may depend on cell type and context.

Iron-dependent lipid peroxidation drives ferroptosis. Excessive accumulation of lipid hydroperoxides alters membrane biophysical properties, leading to a non-pore-forming type of PMR that is distinct from gasdermin- or MLKL-mediated rupture (67). NINJ1 has been shown to be required for PMR during ferroptosis; its oligomerization is triggered by lipid peroxidation products, and *Ninj1* deficiency significantly reduces DAMP release and tissue damage in ferroptosis-associated disease models (63, 68, 69). Mechanistically, lipid peroxidation may alter membrane curvature or generate oxidized phospholipid species that facilitate NINJ1 conformational changes and oligomerization.

PANoptosis is a recently defined inflammatory cell death pathway that cannot be fully explained by pyroptosis, apoptosis, or necroptosis alone (70). It is driven by a multiprotein complex called the PANoptosome, which simultaneously activates the key effectors of multiple death pathways (71). Han et al. further demonstrated that NINJ1 is critical for PANoptosis under pathophysiological conditions, including bacterial infection and heat stress; *Ninj1* deficiency protected mice from lethality and reduced inflammatory cytokine production (72). Given the emerging importance of PANoptosis in infectious and inflammatory diseases, including neurological disorders, understanding NINJ1’s role in this pathway may provide new therapeutic avenues (62).

Mitoxyperilysis is a recently described form of lytic cell death induced by fasting or metabolic stress and is characterized by mitochondrial dysfunction and subsequent plasma membrane rupture (73). Mechanistically, this pathway is driven by mitochondrial reactive oxygen species and calcium influx, which trigger lipid peroxidation-mediated membrane damage, independent of canonical death executors. Notably, while NINJ1 is dispensable for cell death commitment (as *Ninj1* deficiency does not prevent PI uptake and cells still undergo cell death), it is required for efficient plasma membrane rupture, which allows the release of large cytosolic proteins such as lactate dehydrogenase (LDH) and high mobility group box 1 (HMGB1) (73). Thus, in mitoxyperilysis, NINJ1 functions as a downstream facilitator of membrane disintegration rather than an essential executor of the death process itself. Zidan et al. further highlighted this distinction, positioning NINJ1 as a link between metabolic stress and inflammatory outcomes through its role in post-death membrane lysis (61).

Given the diverse roles of NINJ1 in adhesion, inflammation, and PMR, a key question naturally arises: how is NINJ1 activated and regulated? The following section addresses this question.

## 5 Activation and regulation of NINJ1

**Figure 4:**
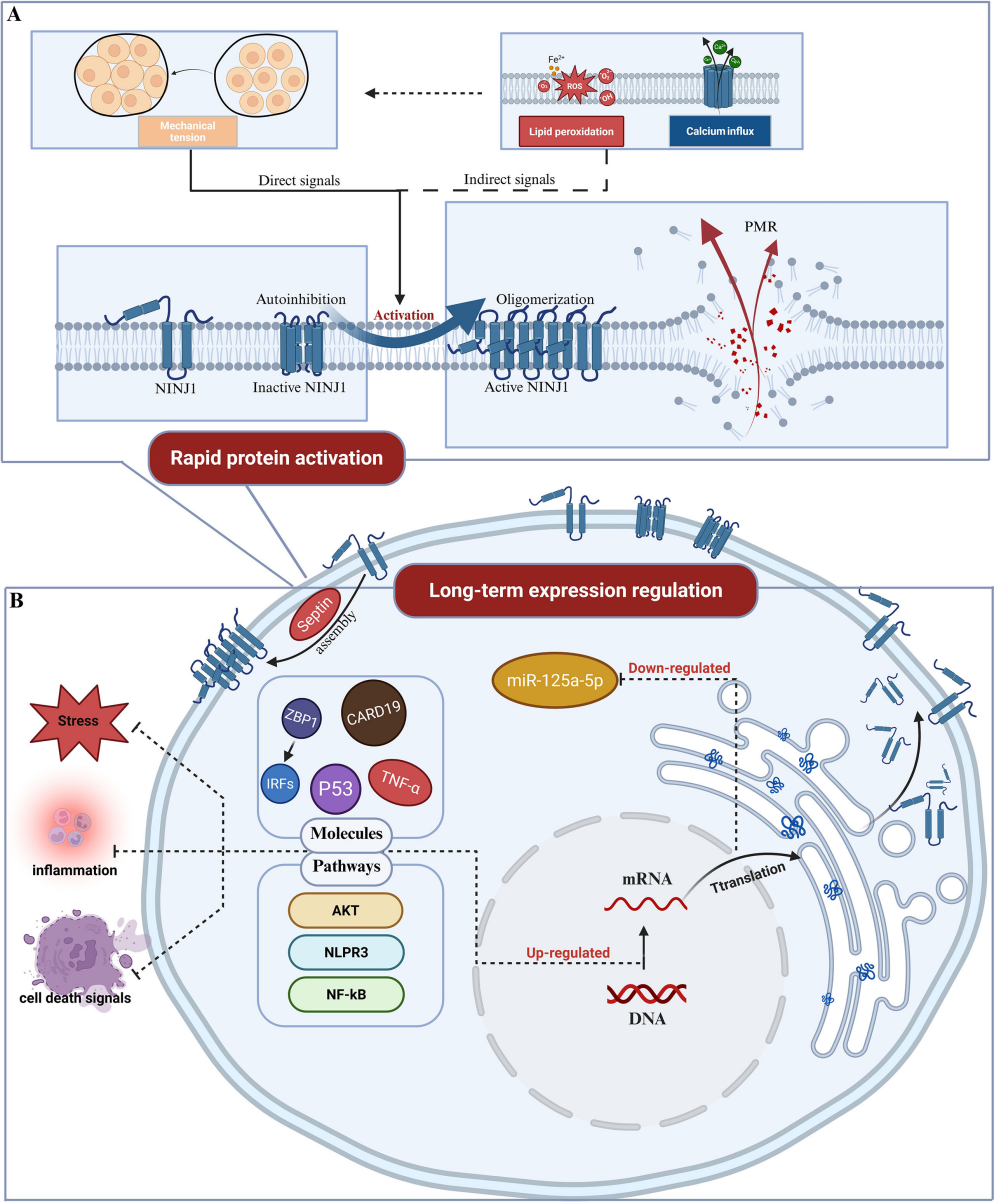
The activation and regulation of NINJ1. It primarily occurs at two levels: rapid protein activation and long-term expression regulation. **(A)** Rapid activation responds to direct triggers such as mechanical tension, as well as indirect signals including Ca²^+^ influx and lipid peroxidation, all of which promote NINJ1 oligomerization. **(B)** Long-term expression regulation involves signaling pathways activated by various stressors and inflammatory factors, encompassing multi−layered regulatory processes from transcription to translation. These two aspects constitute the complex regulatory network of NINJ1. [The image was created using BioRender (https://app.biorender.com/illustrations/6944b378858b39f9d1e07bed?slideId=472692cd-bbb5-4ef4-8d29-a6677d081775)].

The activation and regulation of NINJ1 occur at two interconnected levels: rapid protein activation (conformational changes and oligomerization in response to acute signals) and long-term expression regulation (transcriptional and post-transcriptional control of NINJ1 abundance) (**Figure 4**).

### 5.1 Direct triggers of NINJ1-mediated membrane rupture

Direct signals physically interact with NINJ1 or alter the lipid bilayer to promote NINJ1 conformational changes and oligomerization. Mechanical tension is a well-established, direct activator. During lytic cell death (e.g., pyroptosis), cell swelling generates membrane tension that directly induces NINJ1 oligomerization and PMR (16). The N-terminal domain of NINJ1 is essential, and mutants lacking this domain fail to mediate PMR. Upon sensing physical force, NINJ1 undergoes a conformational switch, exposing its N-terminal domain to interact with adjacent NINJ1 molecules and driving linear polymerization and membrane disruption. Furthermore, NINJ1 can act as a membrane tension sensor (74), lowering the energy barrier for lipid bilayer rupture and making the membrane more prone to damage under mechanical strain. This places NINJ1 not only as an “executioner” but also as a “membrane stability regulator.”

### 5.2 Indirect upstream signals that prime or facilitate NINJ1 activation

Indirect signals do not directly act on NINJ1 but instead modify cellular parameters (e.g., ion homeostasis and membrane lipid distribution) to create an environment permissive for NINJ1 activation.

Calcium (Ca²^+^) influx is a typical example of this phenomenon. During pyroptosis and other cell-death pathways; however, its role is indirect. One proposed mechanism is that Ca²^+^ activates the lipid scramblase TMEM16F, which translocates negatively charged phospholipids (e.g., phosphatidylserine) from the inner to the outer leaflet (17). This loss of lipid asymmetry alters the physicochemical properties of the local membrane environment, indirectly triggering NINJ1 conformational change and oligomerization. The Ca²^+^ chelator BAPTA-AM inhibits NINJ1 oligomerization and stabilizes platelet morphology (43). However, conflicting data exist: Hartenian et al. found that similar cell lysis occurred with or without BAPTA-AM, suggesting Ca²^+^ is not strictly necessary (75). They did observe that Ca²^+^ influx rapidly triggered NINJ1 oligomerization and metabolic death, but this may be explained by Ca²^+^-induced cell swelling rather than a direct NINJ1 interaction. Thus, Ca²^+^ likely acts as an indirect facilitator, not a direct “instructive signal.”

Lipid peroxidation is another well-established upstream signaling pathway that creates a permissive state for NINJ1 activation, particularly during ferroptosis. Ramos et al. demonstrated that ferroptosis inducers promote NINJ1 oligomerization, which is completely blocked by lipid peroxidation inhibitors (e.g., liproxstatin-1). Importantly, NINJ1 itself is dispensable for the initial steps of ferroptosis, including lipid peroxidation. Rather, it acts downstream to initiate the final step of membrane rupture (18). Thus, lipid peroxidation likely acts indirectly by altering membrane biophysical properties (e.g., curvature and fluidity) or generating oxidized phospholipid species that modify NINJ1’s local lipid environment, thereby facilitating its conformational changes and oligomerization.

Taken together, indirect signals such as Ca²^+^ influx and lipid peroxidation do not directly engage NINJ1, but rather remodel the plasma membrane environment, lowering the threshold for its activation. Notably, in resting cells, NINJ1 is maintained in an auto-inhibited face-to-face dimer conformation. Membrane alterations induced by these indirect signals, together with direct mechanical forces, are thought to relieve this autoinhibition, thereby freeing N-terminal domains to initiate oligomerization (76).

### 5.3 Long-term expression regulation

In addition to rapid activation, cells pre-regulate NINJ1 levels and function via a complex network for long-term control. Multiple stress, inflammatory, and cell death signals can significantly upregulate *Ninj1* gene transcription and increase protein expression.

In alcohol-associated liver disease, Z-DNA-binding protein 1 induces the expression of interferon regulatory factors, which bind to the *Ninj1* promoter and directly drive *Ninj1* transcription (77). Under stress, such as DNA damage and calcium overload, p53 directly binds to the *Ninj1* gene promoter region, driving its expression. A bidirectional positive feedback loop forms between them; NINJ1 induced by p53, in turn, stabilizes the p53 protein by inhibiting Mdm2 (a major negative regulator of p53), collectively determining cell fate (78).

Beyond direct transcriptional regulation such as by p53, external factors such as LPS (35, 64), drastic glucose fluctuations (30), Exoenzyme S toxin (79), inflammatory cytokines such as tumor necrosis factor α (80) and important pathways including the NF-κB pathway (81), AKT signaling pathway (32), and inflammasome/caspase-1 pathway (82) have all been shown to indirectly upregulate NINJ1 expression. Furthermore, positive regulators like CARD19 (83) and septin proteins (64) are crucial for the basal expression and structural assembly of NINJ1.

At the post-transcriptional level, NINJ1 expression is strictly inhibited. miR-125a-5p directly binds to the 3′ untranslated region (3′UTR) of *Ninj1* mRNA and inhibits its translation, thereby negatively regulating NINJ1 at the protein level (19). In macrophages, overexpression of this miRNA inhibits LPS-induced inflammatory factor production and monocyte adhesion, exerting anti-inflammatory and vascular-protective effects.

In summary, NINJ1 functions within a complex regulatory network that integrates mechanical and biochemical signals for rapid oligomerization, and its expression is finely tuned by transcription factors, microRNAs, and auxiliary proteins. This multilayered control establishes NINJ1 as a key mediator of cell fate, immune response, and tissue homeostasis.

## 6 Role of NINJ1 in central nervous system diseases

### 6.1 Multiple sclerosis

MS is an autoimmune disorder characterized primarily by inflammatory demyelinating lesions within the CNS, pathologically manifesting as multiple areas of CNS myelin loss, which may be accompanied by damage to neurons and their axons (84–86). The pathological process of MS is heterogeneous and dynamic, involving key events, such as blood-brain barrier (BBB) disruption, inflammatory cell infiltration, microglial activation, gliosis, and axonal degeneration. Ifergan et al. (11) demonstrated that NINJ1 expression was significantly upregulated in myeloid and endothelial cells during EAE. Furthermore, recent discoveries highlighting the role of NINJ1 in cell adhesion and programmed cell death suggest its potential significance in the pathogenesis (11, 87, 88).

Early-stage MS lesions are characterized by peripheral immune cell invasion and BBB breakdown (89). Infiltration of immune cells into the CNS is initiated by their adhesion to the cerebrovascular endothelium. Ahn et al. (10) observed strong NINJ1 expression in myeloid cells (macrophages, monocytes, and neutrophils) and partial expression in endothelial cells (ECs). Furthermore, NINJ1 was shown to enhance adhesion between BV2 cells (a murine microglial cell line of monocytic lineage) and human brain microvascular endothelial cells. Critically, treatment with an NINJ1 antibody significantly suppressed macrophage adhesion to brain microvascular endothelial cells, directly demonstrating its essential role in initiating immune cell extravasation (11). However, adhesion alone is insufficient for immune cells to cross BBB. Ahn et al. (9) discovered that NINJ1 confers strong migratory capacity to immune cells, driving transendothelial migration. NINJ1 overexpression significantly enhanced both random movement and directional transendothelial migration in macrophages, whereas NINJ1 knockdown markedly suppressed these activities. Mechanistic studies revealed that NINJ1 induces the formation of lamellipodia and filopodia at the leading edge of the cell, establishing the dynamic foundation required for migration. More importantly, through its N-terminal domain, NINJ1 activates the key signaling molecule Rac1, thereby initiating the Rac1 pathway to coordinate cytoskeletal reorganization and ultimately enhancing basal motility and transendothelial migration capacity of immune cells. These mechanisms govern the process by which pathogenic immune cells traverse the BBB, leading to CNS infiltration, neuroinflammation, and progression of the MS pathological cascade.

In the chronic phase (90), MS lesions exhibit disrupted iron metabolism and oxidative stress, creating an environment conductive to ferroptosis (91, 92). NINJ1 has recently been demonstrated in several studies to participate in programmed cell death pathways, including pyroptosis and ferroptosis, and to mediate PMR and DAMP release. The released DAMPs can, in turn, activate immune cells, amplify inflammation, and cause myelin damage, thereby establishing a self-perpetuating vicious cycle. However, whether NINJ1 contributes to ferroptosis and DAMP release in the context of MS remains to be established using MS-specific animal models (e.g., EAE) with genetic or pharmacological NINJ1 intervention.

In summary, NINJ1 may play a persistent and multifaceted role throughout the MS pathological cascade (**Table 1**), from mediating immune cell migration across the BBB in early disease to inducing cell death in later stages. Researchers using *Ninj1* knockout mice have observed significantly attenuated EAE clinical symptoms, lower disease scores, and less weight loss compared with wild-type mice. *Ninj1* deficiency ameliorates disease severity by inhibiting inflammatory cell infiltration in the CNS, dampening peripheral immune responses, and modulating chemokine networks (87). Therefore, the development of highly specific CNS-penetrant NINJ1 inhibitors, such as monoclonal antibodies or small molecules, is expected to open a novel therapeutic avenue to control MS progression.

**Table 1:** The function of NINJ1 in various CNS diseases.

Diseases	Biological model	Intervention measure	Consequence	Reference
Multiple sclerosis	EAE rats	/	NINJ1 is upregulated and mediates endothelium adhesion.	(10)
	EAE mouse	Anti–NINJ1 Ab	NINJ1 mediates the migration of myeloid cells to central nervous system inflammatory lesions.	(11)
	EAE mouse	Anti–NINJ1 Ab NINJ1 KO	Knockout or antibody blockade NINJ1 can reduce leukocyte migration and lower the clinical score of EAE.	(87)
Cerebral ischemia	LAA-AIS Patients	/	Elevated circulating levels of NINJ1 were associated with increased risk of LAA-AIS.	(45)
	Rat MCAO Model	/	NINJ1 expression was found to be significantly induced in ischemic brain tissue, mainly in macrophages, neutrophils and endothelial cells.	(25)
	Rat MCAO Model	N-NAM	NINJ1 plays an important role in neutrophils infiltration in the postischemic brain and N-NAM confers robust neuroprotective and anti-inflammatory effects by inhibiting NINJ1 -mediated infiltration of neutrophils.	(97)
	Rat MCAO Model	N-NAM	N-NAM which contains the N-terminal adhesion motif of NINJ1 confers pro-angiogenic effects and suggest that those effects might contribute to its neuroprotective effects in the postischemic brain.	(23)
ICH	Cerebral hemorrhage mice model	NINJ1 KO Atorvastatin	NINJ1 in cerebral hemorrhage can promote the activation of NLRP3, further promoting neuroinflammation. Atorvastatin can interact with NINJ1, inhibiting its mediation of NLRP3 activation.	(5)
TBI	TBI mouse	NINJ1 KO	NINJ1-mediated release of HMGB1 aggravated NETs accumulation by forming a vicious circle following TBI. Knockdown of NINJ1 rescued NETs formation, attenuated BBB leakage, and improved neurological outcomes after TBI.	(6)
SCI	Rat clip compression–induced SCI model	/	NINJ1 expression in the spinal cord is rapidly upregulated, with strong staining appearing in macrophages, microglia, and reactive astrocytes.	(39)
	Mouse contusion SCI model	NINJ1 mAb	Improves locomotor recovery, reduces lesion size, promotes neuronal survival; suppresses pyroptosis-mediated DAMP release and shifts microglial polarization from M1 to M2.	(102)
Neuropsychiatric disorders	Mouse	NINJ1 KO	Ninj1 could be involved in neuropsychiatric disorders associated with impairments of repetitive and anxiety behaviors.	(58)

NINJ1, Ninjurin1; EAE, Experimental autoimmune encephalomyelitis; Ab, Antibody; mAb, Monoclonal antibody; KO, Knockout; LAA-AIS, Large artery atherosclerotic acute ischemic stroke; MCAO, Middle cerebral artery occlusion; ICH, Intracerebral hemorrhage; TBI, Traumatic brain injury; HMGB1, High mobility group box 1 protein; NETs, Neutrophil extracellular traps; BBB, Blood-brain barrier; SCI, Spinal cord injury.

### 6.2 Ischemic stroke and reperfusion injury

Ischemic stroke is a clinical syndrome characterized by neurological dysfunction resulting from disrupted blood supply to the brain tissue, leading to ischemic hypoxic necrosis (93). NINJ1 plays multiple critical roles in this process (**Table 1**).

The occurrence of ischemic stroke involves various risk factors including diabetes, smoking, hyperlipidemia, and hypertension (94), with atherosclerosis as the primary pathological basis. Research has found that under high-risk conditions such as diabetes, oscillating glucose (30) potently induces the upregulation of NINJ1 expression in endothelial cells. Elevated NINJ1 levels can activate the NF-κB pathway or oxidative stress, which in turn upregulates the expression of transendothelial transport proteins such as caveolin-1 (30). This cascade enhances monocyte-endothelial adhesion, initiates vascular inflammation, and ultimately paves the way for the formation and progression of atherosclerotic plaques. Furthermore, patients with large artery atherosclerotic acute ischemic stroke (LAA-AIS) exhibit significantly elevated serum NINJ1 levels, and high NINJ1 levels are independently associated with an increased risk of this stroke subtype (45). However, these data are primarily correlative, and causal inference requires further mechanistic studies using cerebrovascular models.

Following ischemic stroke, a sharp reduction in cerebral blood flow interrupts oxygen and energy supply, triggering excitotoxicity, calcium overload, cytotoxic edema, oxidative stress, and mitochondrial dysfunction (95). The study confirmed that in a transient middle cerebral artery occlusion (MCAO) rat model, NINJ1 expression was significantly upregulated in both the ischemic penumbra and core, strongly suggesting its active involvement in post-ischemic inflammatory amplification and tissue damage (25). Upon blood flow recovery, a complex cascade of reperfusion injury unfolds, often featuring an outbreak of oxidative stress, escalated inflammation, and further disruption of the BBB, leading to additional cell death and expansion of the infarct area (96). During this phase, NINJ1 expression is strongly induced. Studies in a transient MCAO rat model revealed that NINJ1 upregulation is not transient but persists for days after ischemia-reperfusion, with its expression peak coinciding with the peak of neuroinflammation (25). However, these data are primarily correlative, and causal inference requires further mechanistic studies using cerebrovascular models.

Based on NINJ1’s destructive role in mediating inflammatory storms and cell death, blocking specific functional domains may offer therapeutic benefits. Accordingly, researchers synthesized a short 12-amino acid peptide (termed the NINJ1 peptide) based on its cell adhesion region (N-terminal domain). Administration of the NINJ1 peptide in a transient MCAO rat model significantly reduced cerebral infarct volume and improved neurological deficit scores. Further research showed that the NINJ1 peptide markedly reduced infiltration of activated microglia/macrophages and neutrophils into ischemic brain tissue, concurrently lowering pro-inflammatory cytokine levels. It also competitively inhibits homotypic adhesion mediated by NINJ1 between cells (e.g., immune and endothelial cells), alleviates post-ischemic BBB disruption, reduces vascular leakage, and blocks recruitment and migration of immune cells to the ischemic brain, thereby mitigating the inflammatory response and indirectly protecting neurons and the BBB (97).

Despite the dominant destructive role of NINJ1, its functional complexity is reflected in potential repair mechanisms, offering a dialectical perspective for therapeutic strategies. Kim et al. discovered that the NINJ1 peptide could effectively promote angiogenesis *in vitro* and *in vivo* by activating the Ang1-Tie2 and AKT signaling pathways (23). In a cerebral ischemia model, this pro-angiogenic effect significantly increased vascular density and branching within the ischemic brain region, improved cerebral blood flow, ultimately reduced infarct volume, and promoted neurological recovery (23).

Therefore, targeting the specific functional domain of NINJ1 not only alleviates acute inflammatory damage through competitive inhibition but also promotes long-term repair by activating specific signaling pathways, making it a unique therapeutic target capable of intervening across the pathological process of stroke.

### 6.3 Traumatic brain injury

TBI is defined as a disruption of brain function or evidence of brain pathology caused by an external physical force (98). NINJ1, a molecule that responds to physical force and induces cell lysis, plays a pivotal role in both the primary and secondary injury phases (99) (**Table 1**).

The primary mechanical insult of TBI, hematoma compression, and subsequent cerebral edema subject cell membranes to significant physical tension, which directly creates conditions for NINJ1 activation (16). Furthermore, NINJ1 acts as a membrane tension sensor capable of directly perceiving changes in membrane tension and actively reducing lipid bilayer stability through oligomerization (74). Studies in a mouse model of intracerebral hemorrhage (ICH) found that NINJ1 expression was significantly elevated and underwent oligomerization in brain tissue, particularly in the perihematomal area, indicating its activation (5). However, these correlative findings require further validation in TBI-specific mechanistic studies.

Moreover, during the secondary phase of TBI, NINJ1-mediated endothelial cell pyroptosis is a key event in BBB disruption (6). Researchers have observed significant induction of NET formation around cerebral blood vessels in mice after TBI. These NETs promote brain EC pyroptosis via the TLR4/NF-κB pathway, leading to vascular barrier leakage and significant activation of NINJ1 (6). Knockdown of NINJ1 mitigated neutrophil infiltration and NET formation and alleviated NET-mediated brain EC pyroptosis after TBI, thereby reducing BBB destruction and improving neurological outcomes.

In conclusion, NINJ1 plays a crucial bridging role in the pathophysiological process of TBI. Targeting NINJ1 is regarded as a highly promising broad-spectrum neuroprotective strategy, which is expected to intervene in multiple core pathological processes of TBI through a single target.

### 6.4 Spinal cord injury

SCI is a devastating condition that results in permanent motor, sensory, and autonomic dysfunction (100). NINJ1 has emerged as a key player in SCI as evidenced by genetic, expression, and animal studies.

Genetic evidence suggests that NINJ1 variants may influence susceptibility to SCI. A polymorphism in the *Ninj1* gene (rs16878344) was associated with altered risk in a Brazilian cohort, although this finding requires replication (101). In a rat clip compression–induced SCI model, NINJ1 expression in the spinal cord was rapidly upregulated, peaking on day 4 post-injury and then gradually declining. In sham-operated controls, NINJ1 immunostaining was weak in vascular endothelial cells, ependymal cells, and some glial cells, while strong staining was observed in macrophages, microglia, and reactive astrocytes, suggesting its involvement in inflammatory cell responses (39).

Functionally, targeting of NINJ1 has shown therapeutic promise. Yao et al. reported that administration of a NINJ1 monoclonal antibody (mAb) in a mouse contusion SCI model significantly improved locomotor recovery, reduced lesion size, and promoted neuronal survival. Mechanistically, NINJ1 mAb suppressed pyroptosis-mediated DAMP release and shifted microglial polarization from a pro-inflammatory M1 towards an anti-inflammatory M2 phenotype, accompanied by decreased nuclear p-NF-κB p65 and increased p-STAT3 levels (102). Thus, the NINJ1 mAb protects neurons, at least in part, by modulating microglial inflammatory responses.

In summary, NINJ1 is a promising therapeutic target and potential biomarker for SCI. However, most current evidence comes from rodent models; cell type–specific roles (e.g., in astrocytes versus neurons) and the optimal therapeutic window remain to be determined.

### 6.5 Neuropsychiatric disorders

Studies have shown that *Ninj1* knockout mice exhibit significant behavioral abnormalities, including repetitive stereotyped behaviors, increased anxiety-like behavior, and deficits in social interactions (58). Further mechanistic investigations revealed that the absence of NINJ1 led to alterations in the number and activation state of microglia within the prefrontal cortex, a key brain region responsible for higher cognitive function and behavioral control, accompanied by dysregulated expression of synaptic function-related proteins. These findings suggest that NINJ1 plays a crucial role in maintaining proper neural circuit development and homeostasis, potentially through regulation of microglial function (**Table 1**). However, the precise signaling pathways and additional aspects of NINJ1 function in this context require further investigation.

### 6.6 Perspectives on neurodegenerative diseases

Alzheimer’s disease (AD) and Parkinson’s disease (PD) are the most common neurodegenerative disorders, characterized by progressive neuronal loss, protein aggregation (Aβ/tau in AD; α-synuclein in PD), and chronic neuroinflammation. A systematic literature search confirmed that no direct experimental study has examined NINJ1 expression, function, or mechanisms in AD or PD models.

Nevertheless, indirect evidence supports the involvement of NINJ1 in neurodegeneration. The NLRP3 inflammasome pathway is critically implicated in AD and PD, and its activation leads to pyroptosis, which requires NINJ1 for terminal membrane rupture and DAMP release (103). Secondly, NINJ1 participates in ferroptosis-mediated PMR, a process increasingly recognized as a major driver of neuronal loss in both AD and PD (104). Third, a recent hypothesis proposed that the GPR68–NINJ1 axis might serve as a mechanochemical checkpoint in BBB disruption, a common feature of neurodegeneration (105). Collectively, these observations suggest that NINJ1 is a convergent downstream mediator linking neuroinflammatory and cell death pathways to neurodegeneration.

Thus, NINJ1 may act as a downstream effector linking multiple cell death pathways to neurodegeneration; however, this remains untested. Future research should examine NINJ1 expression and function in AD/PD models and patient tissues and explore its therapeutic potential in these conditions.

### 6.7 Cell−type−specific considerations of NINJ1 in the CNS

Most of the current knowledge regarding NINJ1 function is derived from peripheral macrophages, endothelial cells, or non-neuronal models. However, the CNS comprises highly specialized cell types with distinct metabolic and inflammatory profiles, and extrapolating peripheral findings to the CNS without validation remains problematic. Here, we summarize the available evidence and knowledge gaps regarding NINJ1 expression in major CNS cell types.

Microglia are resident immune cells of the CNS and share many features with peripheral macrophages. In the EAE model of MS, NINJ1 is upregulated in microglia/macrophages and promotes their adhesion to brain endothelial cells, a critical step for CNS entry (9, 10). Blockade of NINJ1 with antibodies reduces myeloid cell transendothelial migration and ameliorates disease severity (9, 11). In SCI, NINJ1 expression is strongly induced in microglia, and reactive microglia exhibit NINJ1 upregulation (39). Treatment with an NINJ1 monoclonal antibody shifts microglial polarization from a pro-inflammatory M1 to an anti-inflammatory M2 phenotype, suppresses pyroptosis-mediated DAMP release, and improves functional recovery (102). In a model of chronic cerebral hypoperfusion, hypoxia-induced NLRP3 inflammasome activation in microglia, leading to GSDMD-N and NINJ1 release and subsequent “bystander” neuronal pyroptosis (106). Collectively, these studies suggest that NINJ1 is a key downstream effector of microglial inflammatory responses across diverse CNS pathologies. However, most evidence is derived from bulk tissue analyses or global pharmacological interventions. The specific contribution of microglial NINJ1 to immune cell infiltration remains to be determined, and conditional knockout studies targeting microglia are urgently required.

NINJ1 was originally identified in neurons and Schwann cells following peripheral nerve injury (8). In the CNS, *Ninj1* mRNA is expressed in diverse neuronal populations, including those in the cerebral cortex, hippocampus, cerebellum, and spinal cord (10). In SCI, NINJ1 is also expressed in neurons after injury, and treatment with an NINJ1 monoclonal antibody reduces neuronal loss and promotes survival (102). Notably, the role of NINJ1 in mediating PMR during lytic cell death has not been directly examined in neurons during excitotoxic or ischemic injuries. Although NINJ1 is present in neurons, its functions in most CNS diseases remain largely unknown.

However, no direct studies have examined NINJ1 expression or function in astrocytes, oligodendrocytes, or other glial cell types in CNS disease models. Although indirect evidence suggests a possible role for NINJ1 in these cell types, direct experimental evidence is lacking. This represents a critical knowledge gap and warrants further investigation using cell type–specific knockout tools and single-cell transcriptomics.

## 7 Targeted intervention strategies and therapeutic prospects of NINJ1

Recent advances in understanding NINJ1’s central role in cell death and inflammation have led to the development of intervention strategies targeting its functions. Current research has primarily focused on direct functional blockade, competitive inhibition, and the regulation of upstream expression.

### 7.1 Balancing acute benefits and chronic risks: a critical distinction

Given that NINJ1 also plays essential physiological roles in cell adhesion, tissue repair, and homeostasis (8, 20, 53, 54, 57), it is critical to distinguish between two distinct therapeutic scenarios: acute inhibition to control inflammatory damage versus chronic or systemic inhibition that may disrupt normal tissue functions.

Acute inhibition (e.g., administration of anti-NINJ1 antibodies, N-NAM peptide, or small molecules during the early phase of stroke, TBI, or sepsis) aims to block NINJ1 oligomerization and subsequent PMR, thereby limiting DAMP release, reducing neuroinflammation, and preserving BBB integrity. In these settings, the intervention is time-limited, typically spanning hours to days, and the primary goal is to interrupt the self-amplifying injury cascade. Preclinical evidence supports the safety and efficacy of acute NINJ1 blockade in cerebral ischemia and EAE (87, 97), with no obvious adverse effects reported in the short term.

In contrast, chronic or systemic NINJ1 inhibition (e.g., long-term dosing or genetic deficiency) carries potential risks. NINJ1 knockout mice exhibit increased susceptibility to colitis, microbial dysbiosis, and behavioral abnormalities (57, 58). Moreover, NINJ1 contributes to angiogenesis, myocyte differentiation, and vasa vasorum maturation (23, 52, 54). Therefore, long-term inhibition might interfere with tissue repair, immune homeostasis, and development. For chronic CNS conditions such as multiple sclerosis, or potential future applications in neurodegenerative diseases, intermittent or cell type–specific targeting strategies (e.g., using cell-specific delivery or reversible inhibitors) are preferable to sustained systemic blockade.

This distinction has direct implications for CNS diseases. Thus, acute NINJ1 blockade is a highly promising strategy for ischemic stroke and TBI. In MS, where chronic inflammation persists, intermittent or CNS-penetrant inhibitors with periodic dosing might be considered, while avoiding complete systemic knockout. For future applications in neurodegenerative diseases, a deeper understanding of NINJ1’s role in glial and neuronal cells is required before chronic inhibition can be considered.

### 7.2 Direct functional blockade (monoclonal antibodies)

Kayagaki et al. (107) identified targeted mAbs (e.g., Clone D1/Clone 25) that specifically bind to the N-terminal domain of NINJ1. By inducing steric hindrance, these antibodies effectively inhibit functional oligomerization, thereby preventing PMR. This approach has been validated in various models, including acute hepatitis, ischemia-reperfusion injury, and EAE (108), demonstrating significant reduction in tissue damage and improved disease outcomes.

### 7.3 Competitive inhibition (synthetic peptides)

Synthetic short peptides (e.g., N-NAM) designed based on the N-terminal functional domain of NINJ1 competitively bind to cell-surface NINJ1, effectively blocking NINJ1-mediated homotypic adhesion and subsequent immune cell infiltration (23, 97). In cerebral ischemia models, this strategy mitigates neuroinflammation, protects BBB integrity, and exerts significant neuroprotective effects.

### 7.4 Upstream expression regulation (miRNAs and small molecules)

miRNAs and their mimics (e.g., miR-125a-5p) directly inhibit translation of NINJ1 mRNA, contributing to its anti-atherosclerotic effects (19). Furthermore, small-molecule compounds such as glycine (76, 109), N,N-dimethyl-3β-hydroxycholenamide (DMHCA) (33), as well as drugs such as amlodipine (80) and atorvastatin (5), have been shown to inhibit NINJ1 expression and activation through various pathways, thereby reducing inflammation and improving prognosis. **Table 2** summarizes these potential NINJ1-targeting intervention molecules and their mechanisms of action. Beyond those listed, compounds that interfere with intracellular signals required for NINJ1 activation (e.g., the Ca²^+^ chelator BAPTA-AM) also provide indirect avenues for functional regulation (43).

**Table 2:** Potential intervention strategies and molecular tools targeting NINJ1.

Molecule/drug	Type	Mechanism of action	Models / assays	Reference
CloneD1/Clone 25	Monoclonal Antibody	Binding to NINJ1 prevents these oligomers from forming	Mouse hepatitis model	(107)
NINJ1 mAb	Monoclonal Antibody	Suppresses pyroptosis-mediated DAMPs release; shifts microglial polarization from M1 to M2; decreases nuclear p-NFκB p65 and increases p-STAT3	Mouse contusion SCI model	(102)
Anti–NINJ1 blocking Ab	Antibody	Binding to NINJ1 N-terminal ectodomain decreases the adhesion	EAE mouse	(11)
N-NAM	Short Peptide	Competitively inhibiting NINJ1-mediated homotypic or heterophilic interactions	Rat MCAO model	(23, 97)
NINJ1-B peptides	Short Peptide	Binding to NINJ1 and induces p53 expression	In vitro models (cancer cell lines)	(108)
Glycine	Amino Acid	Inhibiting NINJ1 clustering	In vitro models (various cell death types)	(76)
			Mouse flagellin- induced blood coagulation model	(109)
DMHCA	synthetic steroid	Inhibiting NF-κB pathway, downregulating NINJ1 expression	Mouse retinal Ischemia–reperfusion injury model	(33)
Amlodipine	Drug (Dihydropyridine calcium channel blocker)	Inhibiting NF-κB pathway, downregulating NINJ1 expression	In vitro models (human endothelial cells)	(80)
Atorvastatin	Drug (Lipid-lowering Agent)	Binding to NINJ1 and inhibits its mediation of NLRP3 activation	Mouse cerebral hemorrhage model	(5)
miR-125a-5p	microRNA	Binding to the 3′UTR of NINJ1 mRNA and inhibits NINJ1 expression	In vitro models (Mouse brain capillary endothelial cell 4)/ Mouse diabetes model	(19)

NINJ1, Ninjurin1; EAE, Experimental autoimmune encephalomyelitis; SCI, Spinal cord injury; N-NAM, N-terminal adhesion motif; MCAO, Middle cerebral artery occlusion; DMHCA, N,N-Dimethyl-3β-hydroxycholenamide; NF-κB, Nuclear Factor Kappa B; NLRP3, NLR Family Pyrin Domain Containing 3; 3′UTR, 3' untranslated region.

With the growing understanding of NINJ1’s biological functions and the emergence of more potent and specific inhibitors, targeting NINJ1 holds promise as a novel therapeutic strategy for a range of diseases. However, careful consideration of the balance between acute benefits and chronic risks, as well as cell type–specific and context-dependent roles, is essential for successful clinical translation.

## 8 Conclusion

Since its discovery as a nerve injury-induced adhesion molecule, NINJ1 has been redefined as the central executor of plasma membrane rupture across multiple lytic cell death pathways, positioning it as a key link between cell death and inflammation. The fact that NINJ1 is beneficial for repair but detrimental during inflammation makes it a challenging therapeutic target. In the CNS, NINJ1 contributes to the pathogenesis of MS, ischemic stroke, TBI, and SCI by mediating immune cell infiltration, pyroptosis, DAMP release, and microglial polarization. However, there are significant knowledge gaps. Cell type–specific roles of NINJ1 in the CNS remain largely unexplored. Neurodegenerative diseases (AD and PD) have not been directly studied despite strong mechanistic links via the NLRP3 inflammasome/pyroptosis and ferroptosis.

Therapeutic translation requires careful distinction between the acute beneficial and chronic detrimental effects of NINJ1 inhibition: while short-term inhibition can limit inflammatory damage in conditions such as stroke or SCI, prolonged systemic inhibition may interfere with tissue repair and immune homeostasis. Future research should prioritize cell type–specific knockout models, single-cell transcriptomics of human CNS tissues, and preclinical evaluation of NINJ1-targeting strategies in AD/PD and SCI models. Addressing these knowledge gaps will pave the way for precision therapies that harness or block NINJ1 function in a context-dependent manner.
